# Mitochondria and Antibiotics: For Good or for Evil?

**DOI:** 10.3390/biom11071050

**Published:** 2021-07-17

**Authors:** Juan M. Suárez-Rivero, Carmen J. Pastor-Maldonado, Suleva Povea-Cabello, Mónica Álvarez-Córdoba, Irene Villalón-García, Marta Talaverón-Rey, Alejandra Suárez-Carrillo, Manuel Munuera-Cabeza, José A. Sánchez-Alcázar

**Affiliations:** Andalusian Center for Developmental Biology (CABD-CSIC-Pablo de Olavide University) and Center for Biomedical Network Research on Rare Diseases, Carlos III Health Institute, 41013 Seville, Spain; juasuariv@gmail.com (J.M.S.-R.); carmenj3b@gmail.com (C.J.P.-M.); sulevapovea@gmail.com (S.P.-C.); monikalvarez11@hotmail.com (M.Á.-C.); villalon.irene@gmail.com (I.V.-G.); martatalrey@gmail.com (M.T.-R.); asuacar1@alu.upo.es (A.S.-C.); mmuncab@upo.es (M.M.-C.)

**Keywords:** mitochondria, antibiotics, unfolded protein response, neurodegeneration, cancer, mitochondrial diseases, obesity, diabetes, muscle fatigue, aging

## Abstract

The discovery and application of antibiotics in the common clinical practice has undeniably been one of the major medical advances in our times. Their use meant a drastic drop in infectious diseases-related mortality and contributed to prolonging human life expectancy worldwide. Nevertheless, antibiotics are considered by many a double-edged sword. Their extensive use in the past few years has given rise to a global problem: antibiotic resistance. This factor and the increasing evidence that a wide range of antibiotics can damage mammalian mitochondria, have driven a significant sector of the medical and scientific communities to advise against the use of antibiotics for purposes other to treating severe infections. Notwithstanding, a notorious number of recent studies support the use of these drugs to treat very diverse conditions, ranging from cancer to neurodegenerative or mitochondrial diseases. In this context, there is great controversy on whether the risks associated to antibiotics outweigh their promising beneficial features. The aim of this review is to provide insight in the topic, purpose for which the most relevant findings regarding antibiotic therapies have been discussed.

## 1. Introduction

Mitochondria are highly conserved double-membraned organelles of outstanding importance for cell homeostasis. Although being mostly known for their role in ATP production and oxidative phosphorylation (OXPHOS), mitochondria are biochemical hubs contributing to a wide range of cellular events such as calcium homeostasis [1], detoxification [2], thermogenesis [3], steroidogenesis [4], inflammation [5], cell death [6] or oxidative stress control [7]. According to the well- established Endosymbiotic Theory of Evolution [8], mitochondria evolved from α-proteobacteria that, aiming to escape rising environmental threats, developed a symbiotic relationship with eukaryotic cells’ precursors. Throughout evolution, most of the genetic information of the prokaryotic symbiont was transferred to the nuclear genome of the host, while the information for only a handful of genes remained on small circular DNA molecules, the so-called mitochondrial DNA (mtDNA). The mammalian mitochondrial genome is a closed, double-stranded circular molecule encoding two rRNAs, 22 tRNAs and 13 polypeptides. All its protein products are constituents of the enzyme complexes responsible for oxidative phosphorylation [9,10]. mtDNA molecules replicate unidirectionally from two spatially different origins, the H-strand (O_H_) and L-strand (O_L_) replication origins. Replication starts at O_H_ with the synthesis of a daughter strand and progresses to the L strand to produce a full H-strand circle. Once the replication fork passes the second replication origin O_L_, replication of the L strand initiates and proceeds in a direction opposite to that of H-strand replication [11]. In addition to DNA, mitochondria conserved bacterial-type ribosomes (55–60S ribosomes) [12] to ensure the translation of their genes. The human mtDNA transcription is mediated by two transcription initiation sites, a promoter region, upstream enhancer elements, a transcription factor termed mtTFA [13] and a core RNA polymerase. The transcription factor has the ability to wrap and unwind mtDNA, thus inducing conformational changes required for the core RNA polymerase to access the DNA template and initiate the transcription process. Once the RNA strand is complete mTERF (mitochondrial transcription termination factor), also named mtTERM (mitochondrial transcription terminator), stops elongation by bending the DNA helix and constituting a physical barrier. Interestingly, point mutations in the mtTERM binding site have been identified in patients of the mitochondrial encephalomyopathy MELAS (mitochondrial myopathy, encephalopathy, lactic acidosis and stroke-like episodes) [14], corroborating the importance of this protein for the transcription process.

Given the fact that no intron sequences are present in vertebrate mtDNA and that intergenetic sequences are not abundant, the process of mitochondrial RNA translation is relatively simple and requires only a few enzymes. It starts with the recruitment of the mt-mRNA to the mitochondrial small subunit (mt-SSU), to which is bound by the Mitochondrial Translational Initiation Factor 3 (mtIF-3) and proceeds with the cleavage and maturation of the tRNAs [15]. Then, the mt-mRNA is polyadenylated by a mitochondrial poly(A) polymerase [16] enabling the binding of the small (28S) ribosomal subunit, which moves to its 5′ end thanks to initiation factors such as the Mitochondrial Translational Initiation Factor 2 (mtIF-2) [17]. This protein factor belongs to the GTPase protein family and triggers fMet-tRNA (N-formyl-L-methionyl-tRNA^fMet^) binding to the small ribosomal subunit in the presence of GTP and a template. Subsequently, the elongation process can start. In this step, a ternary complex comprising the Mitochondrial Elongation Factor Tu (mtEF-Tu), GTP and a charged mt-tRNA (which can enter the A-site) assembles. The elongation process takes place along the 3 mitoribosomal sites: the A, P and E sites. The A site is the initial binding site of the tRNA. Then, as synthesis proceeds, the tRNA transitions to the P site, leaving the A site free for the binding of the subsequent tRNA, and finally, moves to the E site, from which detaches from the mitoribosome [18]. If a correct codon-anticodon interaction takes place, the mitoribosome promotes GTP hydrolysis leading to the release of GDP-mtEF-Tu. The GTP-mtEF-Tu complex is restored by the direct interaction of mtEF-Tu with the nucleotide exchange factor mitochondrial translation elongation factor Ts (mtEF-Ts) [19]. Once mtEF-Tu is released, the formation of the peptide bond is catalyzed at the peptidyl transferase centre (PTC) in the mitochondrial large subunit (mt-LSU). Subsequently, the P-site of the mitoribosome is occupied by a deacylated mt-tRNA, and the dipeptidyl-tRNA is located in the A-site. The interaction of the mitochondrial Elongation factor G1 (mtEF-G1) with the mitoribosome alters its structural conformation leading to the release of the mt-tRNA from the P-site, which will be occupied by the dipeptidyl-tRNA [20,21]. Finally, the termination of mitochondrial protein synthesis occurs when the stop codon of mRNA is present in the ribosomal A-site and is decoded by a termination, or Release Factor, RF. However, this process remains obscure due to the limitations associated with the study of mitochondrial translation in vitro.

A large number of antibiotics target the bacterial translational ribonucleoprotein machinery. The majority of these compounds bind to one of the three key ribosomal sites: (1) The peptidyl transferase center (PTC), which connects all functional cores in the ribosome, including the tRNA entrance and exits regions, plus the A-site. This complex is the ribosomal active site, catalyzing peptide bond formation by positioning the reactive partners [22]. These kinds of antibiotics either block tRNA substrate binding or disrupt peptide bond formation by binding to the PTC itself [23]. Chloramphenicol is a well-known inhibitor of this peptide bond formation between the A-site and the tRNA [24]. (2) The peptide exit tunnel, which is a region below the PTC where the newly synthesized peptide chain travels through [25]. This region, next to the 50S subunit, is able to regulate peptide assembly, elongation and termination [26]. Most macrolides, such as erythromycin, bind to this domain impeding the progression of the nascent peptides [27]. (3) The 30S subunit, specifically regions next to A-site, P-site or E-site, in order to prevent translocation [28], initiation [29] or even termination [30]. An example is the tetracycline family which binds near the A-site preventing aminoacyl-tRNA from binding [31]. In this review, we will discuss that most of these antibiotics have an impact on mitochondrial function.

Although mitochondria bear their own genome and count on an exclusive set of proteins, the majority of proteins with mitochondrial function are nuclear-encoded. These proteins are transcribed and translated by the general cellular transcriptional machinery prior to being imported into mitochondria. Once in the organelle, they are folded and assembled with mtDNA-encoded polypeptides giving rise to the mitochondrial complexes and supercomplexes that conform the electron transport chain (ETC) [32].

Their bacterial origin, and the fact that they conserve prokaryotic features such as the 55S or 60S ribosomes, makes mitochondria exceptionally sensitive to antibiotics [33]. Antibiotics are one of the most commonly used pharmaceuticals against bacterial infections and have significantly reduced mortality and morbidity worldwide [34]. There are currently thousands of antibiotics available for clinical use with different action mechanisms. Apart from inhibiting bacterial DNA, RNA and cell wall synthesis, some of them target the ETC to infringe oxidative damage and thus, promote bacterial death [35]. The problem arises when apart from targeting infectious bacteria, antibiotics damage mitochondria or the human microbiome. In fact, there is compelling evidence to suggest that the prolonged use of mitochondria-targeted antibiotics is deleterious for mitochondrial function, leading to increased oxidative stress and cytotoxicity in human cells [36]. Moreover, several studies have demonstrated that antibiotic treatments not only affect negatively the diversity of the human gut microbiome but also alter its resistome and the plasmidome [37]. On top of the medical concerns associated with antibiotics’ abuse, the scientific community currently recommends against extensive use of these pharmaceuticals due to the increasing threat of antibiotic resistance. The emergence of pan-resistant untreatable bacterial strains is already a worldwide crisis. According to the Centers for Disease Control and Prevention (CDC), 16 bacterial strains present an urgent, concerning threat to our society these days [38]. In fact, a study carried out in 2006 in the USA demonstrated that antibacterial-resistant pathogens’ infections were responsible for 99,000 deaths every year, with costs to the national health care system amounting to more than $8 billion dollars [39].

**Figure 1:**
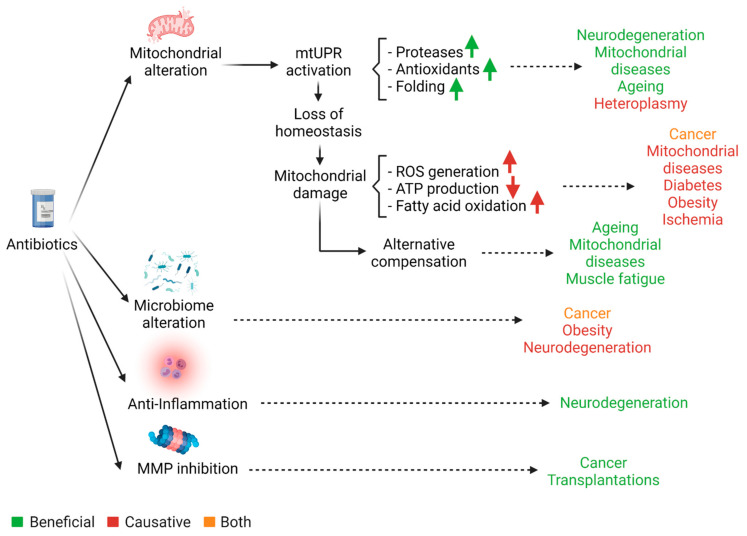
Schematic representation of antibiotics’ general pleotropic effects. Although antibiotics are classically known for their microbicidal or bacteriostatic usage, several studies show that these drugs present numerous pleiotropic effects. Interestingly, these features might represent a potential therapeutic tool, reason why they deserve further consideration and research.

From this standpoint it would seem reasonable to reduce the use of antibiotics in the general clinical practice to cases of utmost importance. Nevertheless, these drugs have proven to be extremely efficacious for the treatment of a wide range of conditions, ranging from cancer to neurodegenerative diseases or ageing. As a matter of fact, recent studies have demonstrated that 5 classes of FDA-approved antibiotics readily target cancer stem cells in different tumor types [40]. Moreover, tetracycline derivatives are being tested as neuroprotectors in synucleinopathies’ models mainly due to their anti-inflammatory properties [41]. This reality contributes to the high controversy on this topic. While a substantial sector of scientists and experts advocates for the strict reduction and thorough regulation of antibiotic use, an increasing number of them is reluctant to ignore their numerous beneficial assets and rather proclaim that the solution to this crisis lies on the generation of new antibacterial agents, namely vaccines. A general overview of the pros and cons associated with antibiotic therapies is presented in Figure 1, in addition to several examples in Table 1.

## 2. Detractors: Adverse Side Effects of Antibiotic Therapies

### 2.1. Mitochondrial Dysfunction & Related Conditions

It is undeniable that antibiotics represent an invaluable asset to fight microbial infections. They have significantly decreased bacterial infection-related mortality rates and contributed to prolonging human life expectancy worldwide. Nevertheless, their use has been linked to numerous adverse side-effects, one of the most common being mitochondrial dysfunction. In fact, it has been demonstrated that most bactericidal antibiotics, irrespective of their specific molecular target, could trigger cell death by acting as stressors that lead to the overproduction of reactive oxygen species (ROS) [42,43] through the disruption of the tricarboxylic acid (TCA) cycle and the mitochondrial ETC [44]. Given the bacterial origin of mammalian cells’ mitochondria, it would not be surprising that antibiotics were targeting both pathogens and the mitochondria from healthy cells. For instance, chloramphenicol reversibly binds to the 50S subunit of the 70S ribosome in both prokaryotic organisms and mitochondria [45], inhibiting peptidyl transferase which catalyzes the two principal chemical reactions of protein synthesis: peptide bond formation and peptide release. In histopathological studies, chloramphenicol-stressed mitochondria showed morphological and functional deterioration [46].

Along this line, a recent study has proven that clinically relevant levels of bactericidal antibiotics cause mitochondrial dysfunction and oxidative stress in mammalian cells in vivo and in vitro. This damage was alleviated when the FDA-approved antioxidant N-acetyl-L-cysteine (NAC) was co-administered. Nonetheless, according to the authors, bacteriostatic antibiotics do not infringe oxidative damage in mammalian cells, even though they are also mitochondria-targeted [47]. It is highly likely that oxidative cellular damage is responsible for the most common side effects associated to prolonged bactericidal antibiotics’ use, ototoxicity [48], nephrotoxicity [49] and tendinopathy [50]. This is especially worrying in patients with compromised antioxidant defense systems or with known genetic risk of developing mitochondrial disorders. Apart from this, several antibiotics can worsen the conditions of individuals bearing mtDNA mutations. Aminoglycosides, for instance, induce ototoxic hearing loss in subjects with mutations in the 12S rRNA gene [51]. Although aminoglycosides typically display specificity toward prokaryotes over eukaryotes, human mitochondrial ribosomes that have A1408 and G1491 at analogous positions exhibit higher resemblance to their bacterial counterparts. This similarity is likely responsible for some of the adverse effects shown by aminoglycosides [23].

Mitochondrial diseases are a set of highly heterogeneous disorders caused by mutations on either nuclear or mitochondrial genes affecting primarily oxidative phosphorylation and ATP synthesis. These conditions are the most common group of inherited metabolic diseases and one of the most common types of neurological disorders [52]. In fact, most mitochondrial diseases’ patients present prominent neurologic and myopathic impairment [53]. It is widely known that neurons have a high energy demand and critically depend on mitochondria to maintain synaptic transmission and structure through the regulation of ATP and calcium levels [54]. Given the fact that antibiotics were reported to impair mitochondrial function in mammalian cells, several studies have focused on assessing the impact of antibiotic treatment on neuronal mitochondria. According to Xiao et al. metronidazole, tigecycline, azithromycin and clindamycin, but not ampicillin or sulfamethoxazole, induced apoptosis in both primary neurons and neuronal cell lines at clinically relevant concentrations. Moreover, they demonstrated that tigecycline, azithromycin and clindamycin triggered cell death through oxidative damage while metronidazole does so in a ROS-independent manner [36]. These results go in line with previous evidence that bactericidal but not bacteriostatic antibiotics induce oxidative stress and damage in mammalian cells [47]. Additionally, tigecycline, azithromycin and clindamycin were reported to be causative of mitochondrial dysfunction in neurons. Interestingly, this effect was abrogated when they were co-administered with an antioxidant.

These findings support the observation that patients given prolonged antibiotic treatments develop neurotoxicity and behavior disorders [55].

### 2.2. Psychiatric Disorders

Certain viral, parasite and severe bacterial infections have been reported to increase the risk for mood and psychotic disorders [56]. Moreover, nationwide population-based studies have proven an elevated risk for a wide range of mental disorders after infections and exposure to antibiotics throughout childhood and adolescence [57,58]. Antibiotic-induced mitochondrial dysfunction might be one of the main factors leading to abnormal behavior conditions [59,60]. Indeed, there is a rising interest in mitochondria on the neuropsychiatric field, since an increasing number of mental disorders have been linked to dysfunctional mitochondria [61,62,63,64]. In this line, the case of ciprofloxacin deserves special mention. This antibiotic interferes with the binding of gamma-aminobutyric acid (GABA) to its receptor. Interestingly, GABA receptor subtype A is regulated by the level of mitochondrial ROS at inhibitory synapses of cerebellar stellate cells [65]. It is therefore believed that ciprofloxacin not only directly inhibits GABA- receptor binding but also deregulates inhibitory GABA-mediated synaptic transmission by damaging mitochondria and triggering ROS overproduction. Presumably as a result of this, a percentage of ciprofloxacin-treated patients develop psychosis [66,67,68].

Nevertheless, the link of antibiotics to psychiatric disorders goes beyond mitochondrial damage. Antibiotic treatments generally disrupt the bacterial microbiota in the intestine and other barriers, which in early developmental stages leads to cognitive changes and depressive-like or anxiety-like behavior in rodents [69,70]. In particular, the gut-brain axis has been described to work through the modulation of brain activity via neural pathways as well as endocrine and immune mechanisms [71,72]. Several studies have been carried out to elucidate whether prolonged or intense exposure to antibiotics at pre-natal stages or early ages increases the risk of psychiatric disorders in humans. A large cohort study in New Zealand established a link between antibiotic exposure in the first year of life and behavioral difficulties combined with mood alterations throughout childhood [73]. Moreover, a recent population study concluded that antibiotic exposure in utero or within the first two post-natal years is associated with increased risk for sleeping disorders, attention deficit hyperactivity disorder (ADHD), conduct and anxiety disorders and other emotional and behavioral conditions with onset in childhood or adolescence. According to the authors these irregularities might arise as an effect of the antibiotic-targeted infections or as a result of antibiotics’ disruption of barrier microbiota leading to opportunistic infections and altered gut-brain axis signaling [74].

### 2.3. Cancer

The association between antibiotic use and cancer development has been highly disputed. Recent studies have provided evidence that antibiotics can influence individuals’ health status by damaging the normal microbiota or microbiome they bear [75]. In fact, variations in gut microbiota have been identified as causative of systemic diseases and dysbiosis with intestinal, metabolic and neurological disorders [76]. It has also been reported that disturbances of the gut microbiota can affect or drive neoplastic conditions. For instance, colorectal cancer (CRC) patients present a clearly distinct fecal bacteria composition to healthy individuals [77]. In particular, the presence of *Fusobacterium* spp. has been closely associated with this type of cancer [78]. These findings point towards antibiotic exposure, and its consequent long-lasting impact on microbiota, being a potential predisposing factor for the development of solid tumors and lymphomas in adult humans. In an aim to ascertain this belief, a recent meta-analysis assessed the results of 25 observational studies on the topic and concluded that, indeed, overuse or prolonged exposure to the main antibiotic classes (beta-lactams, cephalosporins or fluoroquinolones) is linked to an increase of incident cancer diagnoses and lymphomas [79].

Not only do antibiotics increase the risk of cancer development but also seemingly interfere with the action of several oncologic drugs, leading to decreased efficacy and increased toxicity. It is widely known that cancer patients are especially susceptible to infections, either as a consequence of their condition or of treatment-related immune suppression. For this reason, antibiotic consumption among oncologic patients is extremely common and has experienced a notorious increase over time. Gut dysbiosis due to antibiotic exposure has been reported to interfere with immune responsiveness and ultimately, with the success of cancer immunotherapies [80]. In recent years, the use of immune checkpoint inhibitors (ICI) for cancer treatment has been a major breakthrough in the field, especially against solid tumors. However, its efficacy greatly depends on the patient’s immune responsiveness. For this reason, several studies have been carried out to evaluate the impact of antibiotic treatment on cancer patients’ response to immunotherapies and general health status. A retrospective study carried out with 121 renal cell carcinoma and 239 non-small cell lung cancer (NSCLC) patients treated with ICI demonstrated that those who had received antibiotic treatments (generally β-lactams or quinolones) presented an increased risk of primary progressive disease and reduced progression-free and overall survival compared to those that were not exposed to antibiotics [81]. Moreover, the first meta-analysis on the topic, which included 19 studies and 2740 patients, showed that antibiotic use was directly linked to worse overall survival and decreased progression-free in cancer patients treated with ICIs. This negative impact was proven to occur irrespective of the cancer type and the moment of antibiotic treatment application [82].

Apart from disrupting the human microbiome, antibiotics impair mitochondria leading to a profound lack of ATP and elevated ROS levels. Reactive oxygen species are highly mutagenic and have been associated with oncogenic DNA mutations [83]. Moreover, they are responsible for cancer angiogenesis initiation, metastasis and modulation of tumor microenvironment [84]. On top of this, it has been demonstrated that common antibiotics increase lipid peroxide levels and deplete their main antioxidant, glutathione [47]. Lipid peroxidation has been identified as causative of several oncogenic processes, like esophageal carcinogenesis [85], colon cancer [86] or renal cell carcinoma [87]. Additionally, its major product, trans-4-hydroxy-2-noneal is known to preferentially form DNA adducts at codon 249 of the human p53 gene, which is a reported mutational locus in hepatocellular carcinoma [88]. In light of this, it can be inferred that antibiotics are an important factor contributing to carcinogenesis of several, if not multiple cancers via p53 mutation. Moreover, the ability of antibiotics to boost mitohormesis in cancer cells is another factor to consider. Mitohormesis is defined as the paradoxical effect of mild mitochondrial stress that activates cytoprotective mechanisms and leads to enhanced mitochondrial stress resistance [89,90]. The mitochondrial unfolded protein response (mtUPR) is a central effector pathway of mitohormesis and its activation is required for longevity induction in model organisms [91,92]. It has been demonstrated that mtUPR promotes cancer cells’ invasion of tissues [93] and that mitohormetic activation of mtUPR favors tumor metastasis and disease progression [94]. Antibiotics targeting mitochondrial protein synthesis or ATP production are known to infringe stress in mammalian cells, thus boosting the activation of hormetic protective mechanisms such as mtUPR [95]. Thereafter, it is highly likely that prolonged antibiotic exposure potentially favors tumor metastasis and progression via mitohormesis activation in cancer cells. Additionally, the use of specific antibiotics, like chloramphenicol, has already been shown to accelerate cancer progression. Such antibiotic, as well as others that also cause mitochondrial stress and a decrease in ATP biosynthesis, induces matrix metalloproteinase (MMP)-13 expression by activating phosphatidylinositol 3-kinase (PI-3K)/Akt signaling and thus, c-Jun protein phosphorylation. This way, chloramphenicol promotes MMP-13 associated cancer cell invasion [96]. Adverse effects caused by overdose and overuse of chloramphenicol include aplastic anemia, gray baby syndrome, and leukemogenesis [97].

Given the aforementioned evidence and the recurrent use of antibiotics in the common medical practice in our days, several authors have felt the urge to conduct large-scale epidemiological studies to ascertain whether these antimicrobial agents pose a real risk for cancer development. A nationwide cohort study comprising 3,112,624 individuals that was carried out in Finland between 1998 and 2004 concluded that antibiotic exposure is directly proportional to increased risk of a wide range of cancer types. In this way, cancer incidence among individuals with reduced antibiotic exposure was significantly lower than among those conforming the high-exposure cohort. This association was not gender-dependent, the same trend was observed in men and women. The most common cancer types identified in the study were prostate, breast, lung and colon, even though many others were also detected [98].

### 2.4. Obesity

Obesity is a rising topic of concern across developed and developing countries as it is a major risk factor for numerous health conditions such as diabetes, hypertension, cancer, cardiovascular disease, sleep apnea, osteoarthritis, periodontis, atherosclerosis, etc. [99,100]. As reported by the World Health Organization (WHO), worldwide obesity rates doubled between 1980 and 2014, when 13% of the world’s adult population was reported to be obese [101]. Moreover, it is estimated that one third of the global population is nowadays overweight and prone to obesity and related co-morbidities [102]. Efforts are being made to shed light on the factors that have led to this rapid surge of obesity in most countries around the world. Apart from drastic changes in lifestyle, especially concerning diet and activity patterns, more complex and still partially understood contributors must be taken into consideration. One of them being the widespread use of antibiotics.

Commonly used antibiotics elicit different kinds of mitochondrial disturbances. For instance, aminoglycosides target both mitochondrial and bacterial ribosomes [103], quinolones act on bacterial gyrases and mtDNA topoisomerases [104] and β-lactams inhibit cell wall synthesis in bacteria [105] and carnitine/acylcarnitine transporters in mitochondria [106]. Interestingly, several reports have found a clear association between antibiotic exposure during early life and increased risk of obesity in a dose-dependent manner [107,108,109]. This observation was further corroborated by a meta-analysis that evaluated 15 cohort studies comprising 445,880 participants and which concluded that antibiotic exposure is an independent risk factor for childhood obesity [110]. The specific link between the antimicrobial drugs and weight gain remains elusive. Nevertheless, there is compelling evidence to suggest that mitochondrial damage might be the bond between them. In fact, it has been observed that decreased mitochondrial number or impaired mitochondrial function predispose individuals to obesity [111]. Furthermore, differences in mitochondrial enzymes’ activity between obese and non-obese children have been reported, the former presenting a significantly decreased rate of oxidative phosphorylation [112].

Insulin resistance is one of the most common features associated with obesity. In agreement with the works postulating mitochondrial damage as a trigger of obesity, studies have shown that this related condition can also develop as a consequence of mitochondrial malfunction [113]. The increased mitochondrial biogenesis reported during adipocyte differentiation [114] and the implication of mitochondrial dysfunction in disrupted fatty acid oxidation of mature adipocytes [115] hint towards the vital importance of healthy mitochondria for lipid metabolism and support their link it to diet-induced obesity [116]. Furthermore, large populational studies aimed at identifying major DNA variations associated with obesity have revealed that obese individuals share polymorphisms and mutations in mitochondrial related genes. For instance, the single nucleotide polymorphism 15,497 G/A at residue 251 of the MT-CYB gene, which results on aberrant cytochrome b and thus, impaired quinone substrate binding, transmembrane electron transfer and oxidoreductase inhibition [117]. Also mutation of the nicotinamide adenine dinucleotide dehydrogenase subunit I (NDI) gene, encoding a crucial protein for electron transport, is worth being mentioned [118]. Notwithstanding, obesity is a complex condition and the specific mechanisms underlying its induction by antibiotics still need to be clarified. On top of antibiotic-associated mitochondrial dysfunction, antibiotic-driven microbiome disruption has been identified as a potential triggering factor of obesity. In fact, antibiotic alteration of gut microbiota has been reported to affect lipid metabolism leading to this condition [119,120]. Notably, such evidence correlates with the observation of farm animals experiencing a significant weight gain following prolonged antibiotic treatment [121].

### 2.5. Diabetes

The number of adults between 18–99 years suffering from type 2 diabetes mellitus (T2DM) is estimated at 451 million worldwide and thought to increase to 693 million by 2045 if the current trend continues [122]. This unprecedented exponential increase has been explained as a consequence of recent changes in lifestyle, leading to obesity, and lifespan, resulting in an aging global population. On top of this, several experts consider mitochondria as a central hub on this condition. Recent studies have confirmed the link between mtDNA mutations and the development of type 2 diabetes. One of the most common is the mutation m.8561 C>G in the MT-ATP6/8 gene, which encodes subunits of the mitochondrial ATP synthase [123]. Moreover, mitochondrial dysfunction is a key factor on the pathophysiology of T2DM due to its well-established association with insulin resistance [124,125].

Insulin resistance is the hallmark of T2DM etiology. It is described as an attenuated response of metabolically active tissues to insulin, leading to alterations on nutrient fluxes, metabolism and homeostasis. One of the most determinant features of insulin resistance is the excessive accumulation of lipids and their secondary metabolites in metabolically active tissues. Interestingly, mitochondrial dysfunction and the consequent impairment of metabolic fuel oxidation have been reported to drive the accumulation of lipotoxic lipid metabolites. In fact, it has been demonstrated that decreased fatty acid oxidation promotes the synthesis of ceramide and diacylglycerol, which are known to impair the insulin signal transduction pathway [126]. The first hints suggesting a link between insulin resistance and mitochondrial dysfunction came from studies in obese and insulin-resistant individuals who presented reduced skeletal muscle mitochondria oxidative capacity and impaired lipid metabolism compared to healthy controls [127]. These preliminary observations were confirmed by microarray studies that provided evidence of genes controlled by PGC1α and involved in oxidative metabolism being downregulated in the skeletal muscle of T2DM patients [128]. Furthermore, a decrease in mitochondrial respiration was reported among the first-degree relatives of individuals suffering from T2DM, indicating that mitochondrial dysfunction precedes and most likely, triggers the onset of Type 2 diabetes [129].

Mitochondrial dysfunction occurs as a direct result of oxidative phosphorylation or ETC impairment rather than a decrease of mitochondrial content. It is plausible to believe that prolonged antibiotic exposure might result in decreased mitochondrial oxidative capacity, impaired metabolic fuel oxidation and accumulation of lipotoxic lipid intermediates, which culminates in the development of insulin resistance and T2DM [130]. In fact, several studied have reported a link between antibiotic use and increased risk of Type 2 diabetes. A population-based case control study conducted in Denmark comprising 5.6 million individuals concluded that the odds ratio (OR) associating T2DM with exposure to antibiotics was 1.53 (95%confidence interval 1.50–1.55). Interestingly, higher ORs for type 2 diabetes were reported for narrow-spectrum and bactericidal antibiotics compared to broad-spectrum and bacteriostatic types of antibiotics. Moreover, a marked dose-response effect was observed with a rising cumulative load of antibiotics [131]. In line with this, a prospective cohort study including 114,210 women revealed that longer duration of antibiotic use in the previous 4 years was clearly associated with higher risk of diabetes type 2 development. The authors additionally reported that participants who received antibiotic treatments spanning a duration between 15 days and more than 2 months presented a much-elevated risk of T2DM compared with untreated individuals, and therefore advise against the prescription of long-term antibiotic treatments [132].

## 3. Supporters: Antibiotic’s Beneficial Properties

Due to the mitochondrial resemblance to bacteria, antibiotics can have severe side effects on them. However, new perspectives and trends propose that antibiotics could also be used as therapy for many diseases due to their beneficial secondary functions [41,133,134,135]. The advantageous effects of antibiotics have been widely reported in studies carried out in animal and cellular models and additionally in clinical trials, but their specific mechanism of action has not been elucidated yet. The current model explaining antibiotics’ beneficial features is based on their ability to induce a slight disturbance on mitochondrial proteostasis, leading to the activation of a compensatory hormetic effect, where mtUPR plays an essential role [136].

The mtUPR is a mitochondrial stress signaling pathway discovered in mammalian cells [137], but thoroughly studied on *C. elegans* for the sake of simplicity. It is for this reason, that most of what is known about its regulation was discovered in such model organism. Conditions that increase mitochondrial proteotoxicity, an impaired mitochondrial protein quality control machinery, OXPHOS perturbations or increased ROS production are factors that activate the mtUPR [138,139,140]. Several stimuli can lead to these conditions such as elevated temperature, inflammation, toxins, pathogen infections, aging, genetic mutations and antibiotics such as tetracyclines [141]. In *C. elegans*, mtUPR activation pathway is highly characterized, being the Activating Transcription Factor associated with Stress 1 (ATFS-1) transcription factor its main protagonist [138]. On the other hand, the mammalian mtUPR response is more intricated and complex, reason why it is still not well understood. Despite the canonical axis of mtUPR is well stablished in mammals, containing the Activation Transcription Factor (ATF) 4, ATF5 and C/EBP homologous protein (CHOP) proteins [142,143,144], it is still not clear how these regulatory hubs are interconnected with each other or with alternative compensatory pathways such as the endoplasmic reticulum UPR [145,146].

The activation of mtUPR promotes a diverse transcriptional response including the induction of mitochondrial proteases and chaperones, xenobiotic and ROS-detoxifying genes, and metabolic regulators, aimed at recovering mitochondrial function [147,148]. The increase in mitochondrial chaperones such as the matrix-localized Hsp60 and mtHsp70 promotes the folding of recoverable proteins, while the increase of proteases like LONP1 and YME1L removes proteins that fail to re-fold, assemble or which aggregate [149]. Additionally, the mtUPR promotes the expression of anti-oxidant genes including the SIRTs pathway [150], mitochondrial superoxide dismutase and genes involved in glutathione metabolism that limit the protein and membrane perturbations caused by ROS emitted from damaged or altered ETC [138]. Increased transcription of protein homeostasis and anti-oxidant- related genes not only stabilizes the protein-folding environment to promote organelle function but also promotes the recovery or regeneration of salvageable organelles while irreparable ones are degraded via mitophagy [151]. The mtUPR also includes multiple glycolysis genes and lactate dehydrogenase, suggesting that cells may shift to oxidative glycolysis during respiratory chain and mitochondrial dysfunction [92]. This metabolic adaptation would allow cells to generate ATP from a pathway localized at the cytosol and less likely to be affected by mitochondrial dysfunction. Increased glycolysis allows cells to maintain cellular energy levels and therefore be able to carry out normal cellular functions. On top of this, it may also provide the energy required to recover efficient mitochondrial activity. In addition to glycolysis, the mtUPR includes genes related with amino acid and carbohydrate metabolisms suggesting that considerable and complicated metabolic alterations occur during mitochondrial stress [138]. Finally, sets of genes regulating: iron-sulfur cluster biogenesis [152], assembly factors [153], ubiquinone biosynthesis [154], mitochondrial polymerases and dynamics [154,155] are highly likely to play a role on the mtUPR.

Although mtUPR activation looks promising because of the protective function it elicits during mitochondrial exposure to toxins or upon mutation of nuclear-encoded OXPHOS genes, it might be deleterious for cell homeostasis under certain circumstances. In the context of mtDNA heteroplasmy, it helps to maintain and preserve the damaged mtDNA [156]. Moreover, some studies highlight the risks associated with a continuous activation of mtUPR in animal models, such as neurodegeneration or reduced mitochondrial function [157,158]. In conclusion, these two facets of the same pathway indicate that mtUPR induction is potentially a hormetic process: beneficial upon context-specific transient activation but detrimental when chronically activated.

Hormesis is a biphasic dose-response phenomenon characterized by the fact that low doses of a compound have a positive stimulatory effect on a system while high doses can be toxic and inhibitory [159]. In the mitochondrial field, mitohormesis is a term used to define a biological response at which the induction of mild mitochondrial stress leads to an increment in health and viability within cells, tissues or organisms [90], as observed for the antibiotic-induced activation of mtUPR.

### 3.1. Cancer

Cancer is one of the most devastating diseases of our time. Nowadays, there is a broad knowledge about the genomic landscape of cancer, however, it is extremely difficult to identify what are the primary driver mutations in the context of an individual genetic background [160]. In fact, the established hypothesis is that while a few driver-mutations are common to certain specific cancer sub-types, each patient’s tumor is fairly unique in its complexity of genetic changes and that several divergent cancer cell clones may also co-exist, within a single tumor [161]. In the future, personalized medicine will take care of these unique and individual mutations in each patient but meanwhile we need to find new solutions. Tomasetti et al. showed that the life-time risk of two-thirds of cancers could simply be accounted for by the number of times that a given tissue’s stem cells undergo cell division. This is consistent with the idea that during aging, somatic mutations may accumulate in tissue stem cells, driving the formation of cancer stem cells [162]. This correlates with the belief that cancer is essentially a disease of “stemness” gone wrong [163].

Keeping the “cancer stem cell” concept in mind, Lamb and Lisanti et al. showed that 5 different classes of FDA-approved antibiotics can be used to selectively target cancer stem cells, across multiple tumor types [40]. Mechanistically, these antibiotics converge on three main mitochondrial targets: Both mitochondrial ribosome subunits and the mitochondrial oxidative phosphorylation. The tested antibiotics were: Azithromycin, a common macrolide broad-spectrum antibiotic derived from erythromycin; doxycycline, a tetracycline derivative also used as broad-spectrum antibiotic; tigecycline, a new generation tetracycline also called glycylcyclines with a similar mechanism of action and pyrvinium pamoate which is a cyanine dye used as anti-helmintic drug. They demonstrated that these antibiotics were able to inhibit the mammosphere formation (a characteristic 3D cell sphere structure) of several breast cancer cell lines thanks to their ability to interfere with mitochondrial biogenesis. Although stem cells rely mostly on anaerobic metabolism, rather than oxidative phosphorylation, intact mitochondrial function is crucial for their maintenance [164]. Surprisingly, Lamb et al. also demonstrated that there is increased mitochondrial biogenesis in breast cancer stem cells, which is necessary for them to grow as tissue [40]. This increase in mitochondrial abundance and function upon oncogenic transformation was the basis of screening for mitochondrial toxic drugs that selectively inhibit tumor growth [165].

After this discovery, Lisanti et al. refined their potential treatment because, as previously stated, antibiotics also have targets other to tumor cells, thereby affecting healthy cells proliferation and survival. The new formulation was a cocktail containing doxycycline, azithromycin and vitamin C, named DAV [134]. They showed that using this cocktail with low concentrations of antibiotics, cancer stem cells propagation was effectively blocked by ~90% in the breast cancer cell line MCF7. Moreover, the aforementioned tigecycline is gaining relevance in cancer treatment because of its synergistic effect with common chemotherapeutic drugs such as doxorubicin and vincristine [166], venetoclax [133], paclitaxel [167] and cisplatin among others [168]. Antibiotics are a successful therapeutic strategy against cancer for a number of reasons: First, some antibiotics can target DNA non-specifically and have already been approved. Second, since intestinal microbiota modulate the anticancer immune effects of some therapies, such as cyclophosphamide and anti-PD-L1 treatment [169,170], modulation of the fecal microbiome with antibiotics may have a beneficial impact on the outcomes of cancer immunotherapy [171]. Third, since mitochondria play a fundamental role at ATP production and apoptosis regulation [172], antibiotics may modulate cell survival and metabolism of tumor cells by targeting their mitochondria [40].

Overall, future clinical trials for testing the efficacy of mitochondria-targeted antibiotics in multiple cancer types are now clearly clinically warranted. Currently, there are several ongoing clinicals trials related with the potential effect of antibiotics in cancer diseases [173,174,175,176,177,178,179]. Despite the possible side effects of antibiotics, we cannot ignore how promising they are as anticancer therapy [180]. Finally, the bioequivalent dose required to block cancer stem cells and the molecular pathway involved in such process should be assessed carefully, given that prior studies suggest that antibiotics can also affect kinase signaling pathways and the secretion of cytokines, including Interleukin-8 (IL-8), which is known to promote cancer stems cell expansion [181].

### 3.2. Neurodegeneration

Neurodegenerative diseases affect millions of people worldwide, with increasing incidence due to aging population. In this section we will focus on antibiotic-based treatments for various of these disorders. Parkinson’s disease (PD) and dementia with Lewy bodies (DLB) are neurodegenerative conditions characterized by the misfolding and aggregation of alpha-synuclein (αSyn) [34]. The molecular mechanisms involved in synucleinopathies, from αSyn misfolding and aggregation to the numerous cellular effects and pathologies associated remains unclear [182]. Aggregated αSyn disrupts vesicular transport, impairing autophagy, causing mitochondrial dysfunction and affecting vesicle recycling, neuronal plasticity and synaptic integrity [183]. In fact, aggregate forms of αSyn can self-propagate within neurons and to interconnected neural networks throughout the nervous system in a prion-like manner, resulting in the spread of the pathology across the brain and the progression of PD [184]. Several studies have proposed tetracyclines and derivatives, such as doxycycline and minocycline, as neuroprotector antibiotics through several mechanisms: their anti-inflammatory properties [41]; their ability to protect dopaminergic neurons in the substantia nigra [185]; the fact that they regulate the glial response in induced PD animal models [186]; their capacity to both inhibit fibril formation of amyloid beta (Aβ) [187], prion protein (PrP) [188,189], β-microglobulin [190] and aSyn [191] and to block amyloid size and aggregation [192,193].

Minocycline, a tetracycline derivative, presents even more relevant features: it prevents microglial activation without affecting astrogliosis, reduces caspases activation, decreases the activation of nitric oxidase synthases and inhibits glutamate, microglia, nitric oxide-induced neurotoxicity, IL-1 activation, cyclooxygenase-2 expression, and production of prostaglandin E2 [194,195,196]. In a general overview, minocycline has neuroprotective effects in global brain ischemia [197], traumatic brain injury [198], neuronal apoptosis induced by ionizing radiation [199], and reduces cell death in neuronal cultures treated with glutamate [200], and 6-hydroxydopamine- or 1-methyl-4-phenyl-1,2,3,6-tetrahydropyridine (MPTP) -induced nigral dopamine neuron degeneration [201].

Another dramatic neurodegenerative disorder is Hungtinton’s disease (HD), whose pathophysiology remains mostly unknown despite the fact that its driver gene was discovered in 1993 [202]. Currently, the role of neuroinflammantion caused by microglial activation is gaining relevance on the field [203]. As stated before, the immunomodulatory properties of tetracyclines have been clearly demonstrated in various conditions [135]. Paldino et al. showed that administration of doxycycline promotes survival, motor performance, and neuroprotection in mice HD model. These results correlate with a reported significant decrease in the extent of microglial activation [204]. The authors explained the effect of doxycycline through the modulation of CREBs genes, also known as ATFs. The vulnerability of medium spiny neurons of the striatum to HD degeneration is thought to be caused by a transcriptional dysregulation of cAMP and CREB signaling cascades [205]. Paldino et al. also proved that doxycycline increased CREB expression in their model, therefore postulating that preventing a decrease of cAMP signaling and the loss of CREB-regulated gene transcription represents a valid therapeutic strategy for HD [204,206]. The CREB genes family induces transcription of about 4.000 target genes, depending on cell type, stimuli and target [207]. One of the CREBs downstream mediators related to HD is the brain-derived neurotrophic factor (BDNF), a principal neurotrophic factor for both striatal and cortical neurons which is lost but partially recovered after doxycycline treatment [208]. CREB/ATF genes are closely linked to the mtUPR [145,209].

Despite the compelling evidence supporting tetracyclines’ efficacy as a potential treatment for a vast number of neurodegenerative diseases, its exact mechanism of action remains to be elucidated. Moreover, there is a marked discrepancy regarding its administration, dosage and permeability through the blood brain barrier. Nowadays, there are still no clinical trials to shed light on this problem, however, it has been demonstrated that the administration of subantibiotic doses (20–40 mg/day) does not alter bacterial susceptibility to antibiotics while tetracyclines are able to exert the rest of their effects [210]. For these reasons, tetracyclines seem to be a promising therapeutic candidate to treat neurodegenerative diseases.

### 3.3. Mitochondrial Diseases

At first sight, the usage of antibiotics to treat mitochondrial diseases seems paradoxical and controversial because, as previously exposed, antibiotics may interfere with mitochondrial function. Nonetheless, Perry E. et al. performed a 5.000 known bioactive compounds’ screening on MELAS homoplasmic mutant cybrid cells that exhibit complete reduction in respiratory complex function as mitochondrial diseases model, and they discovered that tetracycline antibiotics and inhibitors of the mTOR pathway increased cell survival and fitness [95]. Taking doxycycline as their top compound, they expanded their assays to other mitochondrial mutant cells such as Rieske (complex III) knockout (KO) mouse fibroblasts, mt-ND1 and mt-ND6 homoplasmic mutant cybrid cells, and even with Ndufs4−/− (complex I) KO mice with excellent results. Also, they showed that doxycycline also improved survival in wild-type cells treated with piericidin (complex I inhibitor) or antimycin (complex III inhibitor) during glucose deprivation. Although, Perry E. et al. did not explain the mechanism behind these incredible results, they suggest that the response observed might have been triggered by a doxycycline-induced mitochondrial translation alteration [211] giving rise to a possible “mitohormetic” effect.

In fact, mitohormesis has been closely linked with mtUPR activation [94], metabolite and ion disbalance [212] and ROS production [213]. These mitochondrial stress conditions can trigger extracellular signaling cascades promoting a cell nonautonomous response. Generally, such signaling events involve the production of mitokines, defined as diffusible molecules released from a cell or a tissue in response to mitochondrial stress that induce beneficial responses in other tissues [214]. It has been reported that these stress responses acting through cell nonautonomous signaling were necessary to enhance longevity in *C. elegans* [215]. Mitohormesis can be triggered by various forms of mitochondrial stress that promote a beneficial retrograde signaling response including the modulation of mitochondrial dynamics, the expression of nuclear and mitochondrial-encoded genes as well as of genes related to the antioxidant response, stimulating mitochondrial function and increasing cellular defense mechanisms by either transient metabolic and biochemical alterations or long-lasting cytoprotective mechanisms that increase stress resistance [216].

The activation of this group of compensatory mechanisms has been associated with the regulation of mitophagy and mitochondrial biogenesis as well as with a beneficial mild induction of ROS signaling pathways. Animals presenting impaired cardiac mitophagy and a consequent accumulation of damaged ROS-overproducing mitochondria develop cardiomyopathy. It has been observed that this condition can be improved through the ROS-dependent activation of compensatory mitophagy as a mechanism for mitochondrial quality control that can prevent vicious cycles of ROS formation and mitochondrial dysfunction [217]. Furthermore, muscle-specific knockout mice for COX15, a complex IV assembly protein, are able to express alternative oxidases (AOX) that bypass respiratory complexes III and IV, transferring electrons directly to oxygen, exhibit decreased ROS generation, PGC-1α signaling and lifespan [218]. Livers from adult mice depleted from which mitochondrial superoxide dismutase 2 during embryonic development display mitochondrial adaptive responses with increased mitochondrial biogenesis and antioxidant defenses, while exhibiting decreased ROS [219]. Taken together, these results show the impressive mitochondrial ability to adapt to certain insults.

Currently, there is no treatment for the vast majority of mitochondrial diseases, only palliative therapies. Triggering mitohormesis with antibiotics such as tetracyclines could open a window to a new therapeutic perspective against these pathologies.

### 3.4. Organ Transplant and Ischemia

Donation after neurological determination of death (DCDD) had been the only source of deceased donor organs for most of transplant programs since the late 1960s [220]. Unfortunately, kidney donors are associated with a higher rate of delayed graft function, and reduced graft survival [221]. One of the major issues in organ transplantation nowadays is ischemia, which can be classified in two classes: ischemia at body temperature after loss of vascular flow known as warm ischemia or during the ex vivo storage of grafts/organs in hypothermic conditions known as cold ischemia. Both types of ischemia may lead to metabolic and histologic lesions that are amplified during reperfusion of the organ, potentially resulting in an acute or chronic loss of function [222]. The appearance of these types of ischemia are highly variable between different organs. Whereas warm ischemia at the time of procurement is minimal, there is considerable warm ischemia in retrieved kidneys and this may account for the worse outcomes achieved with these organs.

It has been shown that the accumulation of succinate is a universal metabolic signature of ischemia, and that this drives the accumulation of ROS in the mitochondria during reperfusion [223]. The fact that mitochondria are central to ischemia and/or ischemia–reperfusion injury is supported by several groups studying the protective effects of hydrogen sulfide and carbon monoxide [224]. The mechanism of these interventions appears to be the induction of a hibernation-like state in organs being preserved for transplantation [225]. Using a proteomic approach, Moser M. et al. showed that the levels of several enzymes involved in glycolysis increased when kidneys were cold perfused with solution containing doxycycline, suggesting that the protective mechanism of doxycycline might be a result of increased glycolysis in addition to preservation of mitochondrial structure and function [226].

Further research of Moser M. et al. also demonstrated that metalloproteases (MMP) play a role in injury at the time of cold preservation [227]. In both animal models and human clinical analyses, a considerable release of MMP-2 and MMP-9 as well as injury markers into the preservation solution used in machine cold perfusion has been reported [228]. However, the addition of MMP inhibitors such as doxycycline or MMP-2 siRNA led to a significant decrease in MMP-2 and MMP-9 and injury markers [223]. Moser et al. suggest that doxycycline application prior to cardiac arrest and warm ischemia, was associated with a reduction in injury markers and cellular and mitochondrial injury morphology in a rat model of DCDD kidney preservation. From a clinical point of view, the administration of doxycycline before the withdrawal of life-supporting treatments is a potentially useful intervention that is both simple and non-toxic. In cases of DCDD donation, one must always be wary of the administration of drugs that might bring harm to the donor. It seems unlikely that the administration of this antibiotic would harm the donor, except on the very rare case that the donor has a severe allergy to doxycycline. Also, the administration of doxycycline prior to cardiac arrest would undoubtedly represent a lower risk than the administration of intravenous heparin in high doses, a common practice in many centers that participate in DCDD donation [229]. The administration of doxycycline prior to cardiac arrest might help improve the quality of not only kidneys, but also the heart, lungs, liver, pancreas, and small bowel as well [230]. As a result, there is the possibility that the administration of doxycycline prior to arrest may protect the heart, and may prolong the time before cardiac arrest to beyond the two hours most programs consider an acceptable timespan to wait for asystole [231]. This is certainly a challenge for any pre-cardiac arrest intervention prior to DCDD.

There is increasing evidence to suggest that the structure and enzymatic integrity of mitochondria may be the key to cell surviving preservation or ischemia–reperfusion and that accumulation of succinate may be central to ischemia-reperfusion injury [223]. The mechanism whereby doxycycline, through inhibition of MMP-2, protects the kidney in the setting of cold ischemia appears to function through the protection of mitochondria as well as the preservation of the extracellular matrix. Eight intracellular enzymes have been described to be activated by doxycycline, most of which are involved in glycolysis and in mitochondrial function related to ischemia protection [226]. The administration of an MMP inhibitor before ischemia prepares the cells or their mitochondria to better tolerate the warm ischemic insult, which is not the case when administering the drug after the warm ischemic insult has already occurred [227]. In the long term, this has the potential to increase the quality of transplant kidneys after DCDD and to indirectly increase the number of donor organs available.

Despite their excellent results, Moser et al., suggested that the protective effect of doxycycline on kidneys destined for transplant should ideally be demonstrated with an animal model that tolerates transplantation of the treated and untreated kidneys.

### 3.5. Muscle Fatigue

Muscle fatigue is defined as a decrease in maximal force or power production in response to contractile activity [232], which is a commonly experienced phenomenon that limits athletic performance, restricts daily life and other strenuous or prolonged activity. Currently, there are no established treatments for muscle fatigue. It is known that acute phase protein orosomucoid (ORM) is an endogenous anti-fatigue protein [233]. ORM is predominantly synthesized in the liver, as well as in many extra-hepatic tissues under various physiological and pathological conditions [234]. In fact, ORM is significantly increased in response to various forms of fatigue [235]. Interestingly, ORM could participate in a positive feedback loop to enhance muscle endurance by acting on cell membrane receptor C-C motif chemokine receptor 5 (CCR5) to activate AMP-activated protein kinase (AMPK), increase glycogen synthase (GS) activity, and elevate muscle glycogen content [236]. Therefore, increasing ORM would represent a potential therapy for this disease.

From this standpoint, Wan J. et al. focused-on macrolide antibiotics, which are reported to up-regulate ORM levels [237]. They evaluated several macrolides’ effect and mechanisms in muscle endurance, in order to provide potential treatments for muscle fatigue. They demonstrated that erythromycin, dose-dependently, mitigates muscle fatigue, increases ORM levels and elevates glycogen content in muscle in a mice model [238]. Moreover, they corroborated that erythromycin enhances muscle glycogen and endurance via upregulating ORM and activating ORM-mediated CCR5-AMPK-GS pathway. Studies have shown that AMPK deficiency induces a defect in mitochondrial respiration that appears mainly limited to complex I [239], and that AMPK is a key regulator of mitochondrial biogenesis [240]. Wan J. et al. found that erythromycin treatment on a muscular fatigue mice model remarkably increased muscle complex I activity, ATP levels and mitochondrial biogenesis, presumably in an AMPK-dependent way. For this reason, they proposed erythromycin as a novel drug for therapeutic intervention of muscle fatigue via energy supply improvement.

### 3.6. Aging

There is a clear correlation between mild mitonuclear protein imbalance and extended lifespan [136]. It has been thoroughly demonstrated that prolonged lifespan and improved health in late adulthood can be achieved by partial inhibition of mitochondrial proteins in yeast, worms, fruit flies, and mice [149]. This process can be accomplished by using mitochondrial toxins [241,242] and antibiotics such as tetracyclines [243] or rapamycin [136]. Nowadays, upregulation of the mtUPR has been proposed as a common pathway in lifespan extension induced by mitochondrial defects. To define how intricately mitonuclear protein imbalance and mtUPR are involved in longevity, Houtkooper et al. analyzed its activation in worms exposed to rapamycin [136]. Rapamycin inhibits TOR signaling to alter nDNA translation, inducing mitonuclear protein imbalance, increasing lifespan in various species by inducing mtUPR activation and improving mitochondrial respiration [244,245]. Similarly, the acclaimed lifespan enhancer resveratrol induced mitonuclear protein imbalance, thus activating the mtUPR while increasing respiration and maintaining ATP levels and citrate synthase activity [246,247]. Mitonuclear protein imbalance and mtUPR hence represent an overarching mechanism of longevity that can also be engaged by pathways that signal mainly through the nucleus. Overall, mtUPR seems to result from an imbalance between mtDNA- and nDNA-encoded proteins and is a common feature linking mitochondrial longevity pathways.

The study of hormetic responses has gained considerable relevance since the discovery that mtROS and other mitochondrial stress pathways can promote lifespan in an hormetic-dependent manner [90]. Mitochondrial function declines during aging, contributing to cellular senescence, inflammation and stem cell exhaustion [248]. However, as previously exposed and in concordance with the concept of hormesis, mild mitochondrial damage can be beneficial to cells and extend organismal lifespan, whereas severe stresses can cause irreparable damage. Although the activation of the mtUPR and its function as a cryoprotective stress response is well accepted [249], its role as lifespan inductor is still not entirely clear [250].

## 4. Conclusions

Mitochondria and antibiotics have always been controversial for plenty of reasons, as discussed in this review. However, not all antibiotics function in the same way nor at the same dosage. In fact, inadequate drug dosage and the consequent associated adverse reactions are estimated to be between the fourth and sixth leading cause of death in the USA and an important reason for hospitalization, meaning increased health care costs [251,252,253]. To handle this situation, Pacheu-Grau et al. proposed the standardization of mitochondrial antibiograms to obtain something similar to individualized “barcodes” for antibiotic therapy, which might help avoid antibiotics’ side effects and enable appropriate personalized medicine [254,255]. The variety in antibiotics’ side effects towards mitochondria could be explained by mitochondria-related single nucleotide polymorphisms (SNP). The number and frequency of similar, detrimental mitochondrial SNPs that on a regular basis go unnoticed could be remarkably high. These SNPs could possibly be phenotypically neutral in the absence of antibiotics, inducing a negative selection against them once antibiotic usage starts [256]. Still, we cannot ignore the pleiotropic effects of these drugs because as seen in chemotherapy, the overall outcome of applying them could be beneficial for the patient. In fact, the rising interest on antibiotics as unconventional treatment is explained by the need of new therapies for non-treatable diseases such as mitochondrial diseases or neurodegeneration.

In human physiology, there is still no “magic bullet” as proposed by Paul Ehrlich more than a century ago [257]; every drug or treatment will have a consequence in some way. However, new approximations and perspectives have to be evaluated in order to advance against the numerous incurable diseases that still threaten our society. Antibiotics will not be a panacea, but until we find a better therapy, we should think about them as an alternative and an additional option for patients to choose from. As Paracelsus said “dosis sola facit venenum”.

**Table 1:** Most studied antibiotics considering their mitochondrial benefits and adverse effects. Dosages were retrieved from *Medscape*.

Antibiotic	Type	Pros (in Addition to Their Antibiotic Function)	Mitochondrial Alterations	Max Dosage (mg/Day)	Long Term Treatment (mg/day)
Tetracycline	Tetracycline	Neuroprotection [41,185,186,187,188,189,190,191,192,193]. mtUPR activation [95,141] Inhibit cancer proliferation [40,134] Mitochondrial protection from ischemia [223,227]	Promote heteroplasmy [156] Mitochondrial biogenesis impairment [258] Mitochondrial respiratory chain activity reduction [259] Mitochondrial protein synthesis inhibition [211]	1000	Sub-antibiotic dosage: 20–40
Doxycycline	Tetracycline			200	100
Minocycline	Tetracycline			200	100–200
Tigecycline	Tetracycline			200	100
Azithromycin	Macrolide	Inhibit cancer proliferation [40,134] Mitigates muscle fatigue [238] AMPK activation [238]	Mitochondrial membrane potential disruption [36] ROS production [260] Cytochrome c release [261]	500	250
Erythromycin	Macrolide			4000	500
Rapamycin	Macrolide	Lifespan extension [244,245] mtUPR activation [246,247] Autophagy flux enhancer [262] Increases mitochondrial network fitness [263,264]	Immunosuppression [265] Mitochondrial dynamics alteration [266]	4–6	1–2
Pyrvinium pamoate	Anthelmintic	Inhibit cancer proliferation [40,267]	Mitochondrial respiration inhibition [268]	1000	11 per kilogram

## Author Contributions

J.M.S.-R. and C.J.P.-M. wrote the manuscript; M.Á.-C., I.V.-G., M.T.-R., A.S.-C. and M.M.-C. contributed to the literature search; S.P.-C. and J.A.S.-A. revised the manuscript. All authors have read and agreed to the published version of the manuscript.

## Data Availability

The study did not report any data.

## Abbreviations

ADHD	Attention Deficit Hyperactivity Disorder
AMPK	AMP-activated protein kinase
AOX	Alternative oxidases
aSyn	Alpha synuclein
ATF	Activation Transcription Factor
ATFS 1	Activating Transcription Factor associated with Stress 1
BDNF	Brain-derived neurotrophic factor
CCR5	C-C motif chemokine receptor 5
CDC	Centers for Disease Control and Prevention
CHOP	C/EBP-Homologous Protein 10
CRC	Colorectal cancer
CREB	CAMP Responsive Element Binding Protein
DCDD	Donation after neurological determination of death
ETC	Electron Transport Chain
FDA	US Food and Drugs Administration
fMet-tRNA	N-formyl-L-methionyl-tRNA^fMet^
GABA	Gamma-aminobutyric acid
GS	Glycogen synthase
HD	Huntington’s diseases
HSP	Heat Shock Protein
ICI	Immune Checkpoint Inhibitors
IL	Interleukin
MELAS	Mitochondrial myopathy, encephalopathy, lactic acidosis, stroke-like episodes
MMP	Matrix Metalloprotease
MPTP	6-hydroxydopamine- or 1-methyl-4-phenyl-1,2,3,6-tetrahydropyridine
mTERF	Mitochondrial Transcription Termination Factor 1
mtDNA	Mitochondrial DNA
mtEF-G1	Mitochondrial Elongation Factor G1
mtEF-Tu	Mitochondrial Elongation Factor Tu
mtEF-Ts	Mitochondrial Elongation Factor Ts
mtIF-2	Mitochondrial Translational Initiation Factor 2
mtIF-3	Mitochondrial Translational Initiation Factor 3
mtLSU	Mitochondrial Large Subunit
mtSSU	Mitochondrial Small Subunit
mtUPR	Mitochondrial Unfolded Protein Response
mtTERM	mitochondrial transcription terminator
NAC	N-acetyl-L-cysteine
NDI	Nicotinamide adenine dinucleotide dehydrogenase subunit I
ORM	Orosomucoid
OXPHOS	Oxidative phosphorylation
PD	Parkinson Diseases
PI-3K	Phosphatidylinositol-3 Kinase
PGC-1α	Peroxisome proliferator-activated receptor gamma coactivator 1-alpha
PrP	Prion protein
PTC	Peptidyl Transferase Center
ROS	Reactive Oxygen Species
SIRT	Sirtuin
SNP	Single Nucleotid Polymorphism
T2DM	Type 2 Diabetes Mellitus
TCA	Tricarboxylic Acid
TOR	Target of Rapamycin
WHO	World Health Organization
